# Experimental and Theoretical Studies on Oxidation of Cu-Au Alloy Surfaces: Effect of Bulk Au Concentration

**DOI:** 10.1038/srep31101

**Published:** 2016-08-12

**Authors:** Michio Okada, Yasutaka Tsuda, Kohei Oka, Kazuki Kojima, Wilson Agerico Diño, Akitaka Yoshigoe, Hideaki Kasai

**Affiliations:** 1 Department of Chemistry, Graduate School of Science, Osaka University, 1-1 Machikaneyama-cho, Toyonaka, Osaka 560-0043, Japan; 2 Department of Applied Physics, Graduate School of Engineering, Osaka University, 2-1 Yamadaoka, Suita, Osaka 565-0871, Japan; 3 Center for Atomic and Molecular Technologies, Osaka University, 2-1 Yamadaoka, Suita, Osaka 565-0871, Japan; 4 Synchrotron Radiation Research Center, Quantum Beam Science Directorate, Japan Atomic Energy Agency, 1-1-1 Kouto, Mikazuki-cho, Sayo-gun, Hyogo 679-5148, Japan; 5 National Institute of Technology, Akashi College, Akashi, Japan, 679-3 Nishioka, Uozumi-cho, Akashi-City, Hyogo-Prefecture 674-8501, Japan

## Abstract

We report results of our experimental and theoretical studies on the oxidation of Cu-Au alloy surfaces, viz., Cu_3_Au(111), CuAu(111), and Au_3_Cu(111), using hyperthermal O_2_ molecular beam (HOMB). We observed strong Au segregation to the top layer of the corresponding clean (111) surfaces. This forms a protective layer that hinders further oxidation into the bulk. The higher the concentration of Au in the protective layer formed, the higher the protective efficacy. As a result, of the three Cu-Au surfaces studied, Au_3_Cu(111) is the most stable against dissociative adsorption of O_2_, even with HOMB. We also found that this protective property breaks down for oxidations occurring at temperatures above 300 K.

Copper (Cu) and gold (Au) form model binary metallic systems^1^,^2^,^3^,^4^,^5^,^6^ with stable *L*1_0_ (CuAu) and *L*1_2_ (Cu_3_Au and Au_3_Cu) structures. Experimental studies report surface segregation of Au in Cu-Au alloys^7^,^8^,^9^,^10^,^11^,^12^,^13^,^14^,^15^,^16^,^17^,^18^,^19^,^20^,^21^. More recently, extensive studies^4^,^10^,^11^,^12^,^13^,^14^,^15^,^16^,^17^,^18^,^19^,^22^,^23^ on the oxidation of Cu_3_Au surfaces found Au-rich top-most layers of Cu_3_Au(100), (110), and (111) surfaces. Oxidation, which is one of the more important corrosion process, induces changes in segregation^10^,^11^,^12^,^13^,^14^,^15^,^16^,^17^,^18^,^22^,^23^,^24^,^25^,^26^. Studies have been done to induce Cu segregation to the surface by dissociatively adsorbing energetic O_2_^10^,^11^,^12^,^13^,^22^,^23^. However, even after prolonged doses of 2.3 eV hyperthermal O_2_ molecular beam (HOMB), there were no obvious Cu_2_O growth observed on (100) and (111). These results suggest that alloying of Cu-based materials with Au works as an efficient protection against oxidation into the bulk^10^,^11^,^23^. On the other hand, on the more open (110), additional oxidation processes induced by the enhanced diffusion of constituent atoms from and/or into the bulk contributes to Cu_2_O formation. Although Cu segregation on the surface occurs in a similar way^12^,^22^.

Au segregation depends on the bulk chemical potentials of Au and Cu, i.e., bulk stoichiometry. Thus, we expect to be able to control Au surface segregation, i.e., the Au layer profile on the surface region, by changing the bulk Au concentration^13^,^23^. Herein, we report the results of our detailed studies on the Au layer distribution of Cu_3_Au(111), CuAu(111) and Au_3_Cu(111). We also demonstrate the protective function/nature of such surfaces against oxidation processes induced by energetic O_2_. Increasing Au bulk concentration hinders oxidation. Clean Au_3_Cu(111) contains ca. 100% Au atoms in the first and second layers. Thus, as expected, Au_3_Cu(111) is inert to oxidation. Theoretical studies also support the same conclusion with regard to the protective nature of the surface. However, even on such initially inert surfaces, protection against oxidation fails for processes occurring at higher temperatures.

## Experimental & Theoretical Methodology

To characterize the corresponding surfaces, we use X-ray photoemission spectroscopy (XPS) measurements in conjunction with synchrotron radiation (SR). All experiments were performed using the surface reaction analysis apparatus (SUREAC 2000), constructed in BL23SU at SPring-8^27^,^28^. Briefly, the surface reaction analysis chamber is equipped with an electron energy analyzer (OMICRON EA125) and a Mg/Al-*α* twin-anode X-ray source (OMICRON DAR400). A quadrupole mass spectrometer, which was used to analyze the molecular species in the HOMB, is located opposite to the HOMB source. The base pressure of the surface reaction chamber was below 2 × 10^−8^ Pa. The Cu_3_Au(111) (Surface Preparation Laboratory, SPL), CuAu(111) (MaTeck), and Au_3_Cu(111) (SPL) samples were cleaned by repeatedly sputtering with 1.0~1.5 keV Ar^+^ and annealing at 720 K for 30 min, until impurities can no longer be detected by SR-XPS, and corresponding sharp (1 × 1) LEED pattern observed except for the CuAu(111). The clean CuAu(111) revealed a dim (1 × 1) LEED pattern, suggesting a low bulk crystallinity.

We control the kinetic energy of the incident HOMB by changing the O_2_, He, and/or Ar gas mixing ratios. The corresponding nozzle temperatures used to produce 0.5 and 2.3 eV HOMB are 300 and 1400 K, respectively. A typical flux density at the sample position in the present experiments would be ca.10^14^–10^15^ molecules ⋅ cm^−2^ ⋅ s^−1^, at HOMB kinetic energies of 0.5 and 2.3 eV. After irradiating the Cu_3_Au(111) with the desired amount of HOMB normal to the surface, we measured the corresponding high-resolution SR-XPS spectra at 0°, 35°, and 70° from the surface normal, using a monochromatic SR beam with a photon energy of 1090.5 eV.

We also performed density functional theory based total energy calculations^29^,^30^, within the Generalized Gradient Approximation (GGA)^31^, using plane waves (600 eV cutoff energy) and pseudopotentials^32^. We model CuAu(111) and Au_3_Cu(111) using periodic slabs. Each slab has seven fcc (111) layers, separated by ca. 1.50 nm of vacuum, repeated in a supercell geometry, with dipole correction applied. Each layer in the slab contains four atoms, so that the composition (of Au) can be varied in steps of 25%. We have chosen sufficiently large supercells so as to avoid interaction between the O(O_2_) in neighboring supercells. We performed Brillouin zone integration using the Monkhorst-Pack special point sampling technique^33^, with 9 × 9 × 1 sampling meshes. The bottom four layers comprise the unsegregated layers having bulk geometry, i.e., 50%–Au 50%–Cu in the *L*1_0_ ordered structure (CuAu) and 75%–Au 25%–Cu in the *L*1_2_ ordered structure (Au_3_Cu). The bottom four layers were kept fixed to the corresponding optimized theoretical bulk lattice constants, viz., *a* = 0.394 nm, *c* = 0.365 nm (CuAu), and *a* = 0.394 nm (Au_3_Cu). The top three layers, viz., the first surface layer, the second-, and third- (sub-surface) layers, comprise the segregated layers and allowed to relax. We also carried out similar calculations for bulk Cu, bulk Au, bulk CuAu, bulk Au_3_Cu, and O_2_.

## Results and Discussion

### Au Segregation and Concentration Profile

**Figure 1:**
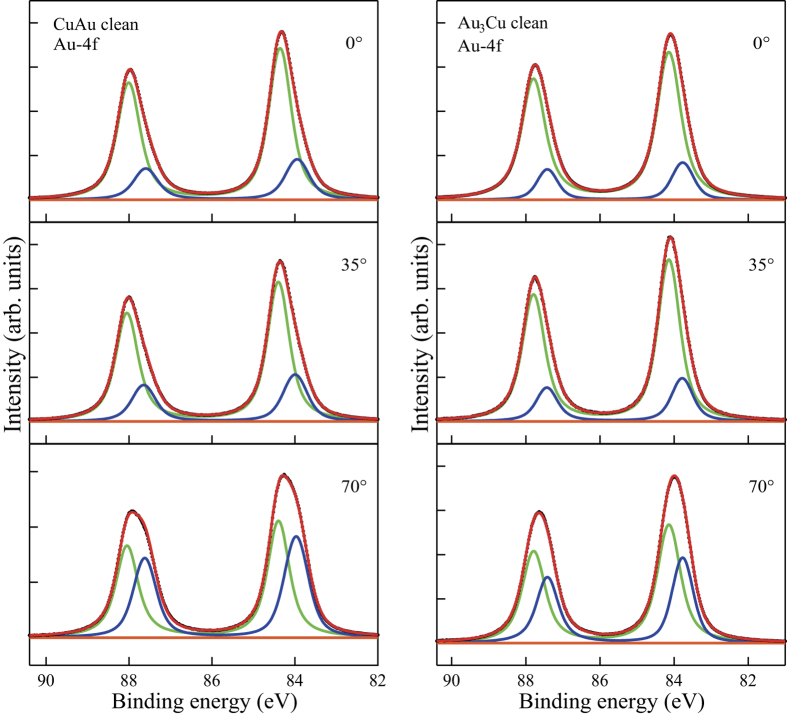
Detection angle dependence of Au-4f SR-XPS spectra on clean CuAu(111) (left panel) and Au_3_Cu(111) (right panel). Surface normal detection: 0°. Oblique detection: 35° and 70°. The XPS spectra can be clearly separated into bulk (B) and surface (S) components, green and blue lines, respectively (see also text). The S components peak at relatively lower binding energies than that of the corresponding B components. The background was already subtracted by the Shirley method^34^. Intensities given in arbitrary units and intensity scales differ between panels (i.e., differ between samples and detection angles).

**Figure 2:**
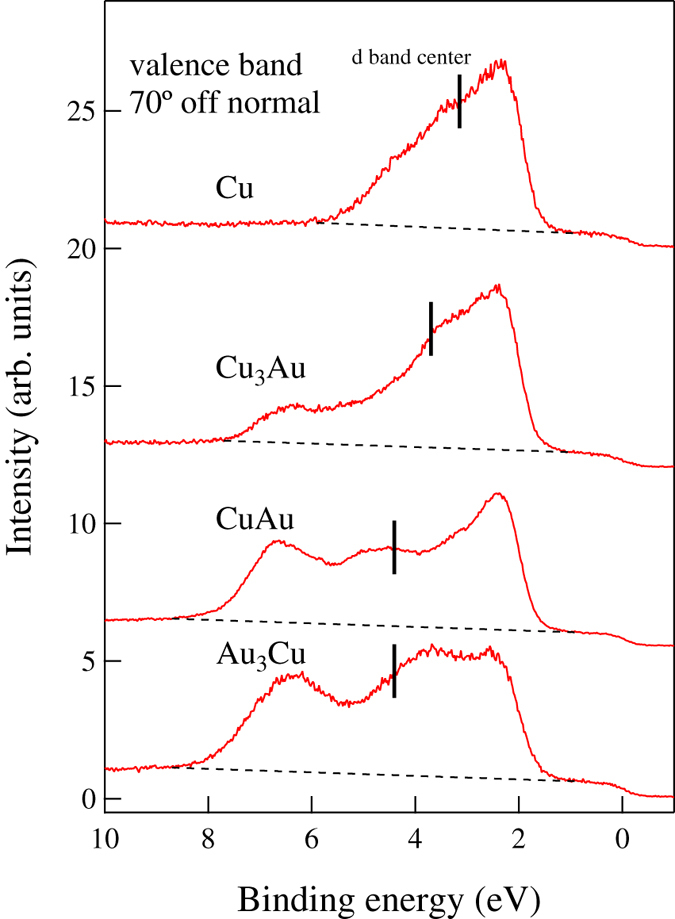
Bulk Au concentration dependence of valence band SR-XPS spectra of clean Cu(111), Cu_3_Au(111), CuAu(111), and Au_3_Cu(111). Red and black lines show the peak curves and the linear fitting (for the *sp*-band contribution and background, both of which need to be subtracted from the spectra) used in determining the location of the *d*-band center, respectively. The short vertical lines on each peak indicate the positions of the corresponding *d*-band centers, viz., Cu: 3.1 eV, Cu_3_Au: 3.7 eV, CuAu: 4.5 eV, and Au_3_Cu: 4.4 eV, respectively, for detection 70° from the surface normal. For detection along the surface normal, the *d*-band center are located as follows: Cu: 3.2 eV, Cu_3_Au: 3.6 eV, CuAu: 4.4 eV, and Au_3_Cu: 4.5 eV.

**Figure 3:**
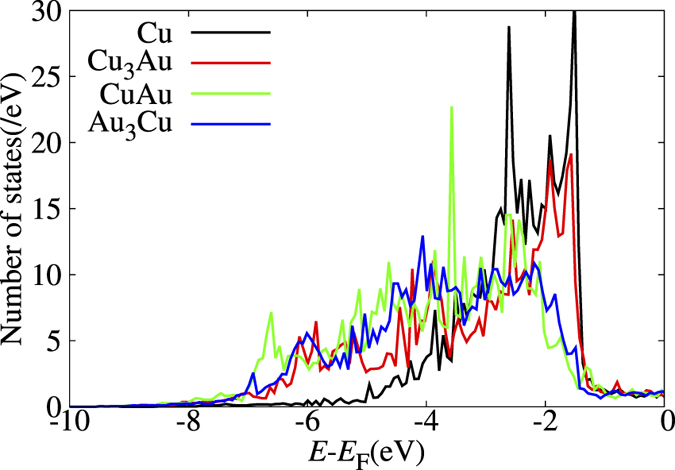
Projected density of states of atoms on the top layer of clean Cu(111) (black line), Cu_3_Au(111) (red line), CuAu(111) (green line), and Au_3_Cu(111) (blue line). The corresponding *d*-band centers are located as follows: Cu: −2.45 eV, Cu_3_Au: −2.86 eV, CuAu: −3.37 eV, and Au_3_Cu: −3.24 eV, respectively. Energies given in [eV] with respect to the Fermi level (*E*_F_). (Calculated binding energy *E*_B_ = |*E − E*_F_|).

In Fig. 1, we show the Au-4f SR-XPS spectra of clean CuAu(111) and Au_3_Cu(111), measured at 0°, 35°, and 70° from the surface normal. (For the Cu_3_Au(111) results, cf., ref. 13). The Au-4f XPS spectra were fitted with the Voigt function, defined as the convolution of a Lorentzian with a Gaussian line shape. The background was subtracted by the Shirley method^34^. Similar to Cu_3_Au(100)^10^,^11^,^35^, Cu_3_Au(110)^12^, and Cu_3_Au(111)^13^, we can clearly separate both the Au-4f_7/2_ and Au-4f_5/2_ XPS peaks into bulk (B) and surface (S) components. The B components peak at binding energies *E*_B_ = 84.48 eV, 84.39 eV, 84.14 eV (Au-4f_7/2_) and *E*_B_ = 88.10 eV, 88.04 eV, 87.79 eV (Au-4f_5/2_) for Cu_3_Au(111), CuAu(111) and Au_3_Cu(111), respectively. The S components peak at relatively lower *E*_B_ than that of the corresponding B components. We see that, consistent with previous reports^35^,^36^, the *E*_B_ peak positions of the B components increase with increasing bulk Au concentrations. Also, consistent with Au-rich termination, we can observe clear Au-4f surface core-level shift (SCLS) in Fig. 1. The corresponding SCLS values are Cu_3_Au(111): −340 meV; CuAu(111): −420 meV; and Au_3_Cu(111): −370 meV. For comparison, the SCLS for pure Au(111) is −350 meV^37^. The difference in surface coordination of the Au atoms (vide ante) accounts for the bulk Au concentration dependence of SCLS. Reduced coordination leads to narrower valence bandwidth. Narrowing of the bandwidth, in turn, increases the density of states. To maintain a common Fermi level, charge must flow between the surface atoms and the bulk. If more than half of the valence band is filled, the surface narrowed band center is lower than the bulk Fermi level and the binding energy decreases. In contrast, if less than half of the valence band is filled, the surface narrowed band center is higher than bulk Fermi level, and the binding energy increases. This explains the trends observed experimentally, as corroborated by the measured valence band spectra and the corresponding calculated density of states (cf., Figs 2 and 3).

We determined the layer Au concentration profile from the detection angle dependence of the Au-4f XPS peak intensity, which has B, S, and I (interface layer) components. We can approximate the peak intensity ratio of S to B (*A*_S_/*A*_B_) for a clean Cu_3_Au surface by the following simple equation,


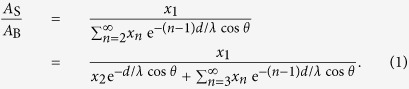


*x*_*n*_ gives the Au fraction of the *n*-th layer from the surface. *d* gives the interlayer distance. The corresponding Au-4f photoelectron mean free paths *λ* in each Cu-Au alloy can be obtained following previous studies^38^. For Cu_3_Au, CuAu, and Au_3_Cu, we get *λ* = 1.48, 1.56, 1.63 nm, respectively (cf., Supplementary Information). *θ* is the photoelectron detection angle from the surface normal. From *A*_S_/*A*_B_ measured at *θ* = 0°, 35°, and 70°, we obtain *x*_1_ and *x*_2_ for the clean surface, assuming *d* to be the bulk interlayer distance, ignoring layer relaxation, and taking *x*_*n*≥3_ to be the bulk value.

For Cu_3_Au(111): *d* = 0.217 nm, *x*_*n*≥3_ = 0.25; thus giving us *x*_1_ = 0.51 and *x*_2_ = 0.32.

For CuAu(111): *d* = 0.225 nm, *x*_*n*≥3_ = 0.50; which gives us *x*_1_ = 0.94 and *x*_2_ = 0.72.

For Au_3_Cu(111): *d* = 0.230 nm, *x*_*n*≥3_ = 0.75; and *x*_1_ = 1.00 and *x*_2_ = 1.00.

These values agree well with our theoretical predictions, as shown in Table 1. Surprisingly, the first and the second layers of Au_3_Cu(111) contain almost 100%-Au atoms. This indicates that Au_3_Cu(111) would be inert (to oxidation).

In Figs 2 and 3, we show the measured valence band spectra and calculated sum of the projected density of states (PDOS) of atoms on the top layer of the clean Cu(111), Cu_3_Au(111), CuAu(111) and Au_3_Cu(111), respectively. The evolution of Au-related features correspond to the Au-segregated layer profile in Table 1. The *d*-band of surface atoms and *d*-band centers are shifted to lower energy levels (corresponding to increasing binding energies) with increasing Au concentration on the surface, with a maximum for CuAu(111), consistent with the SCLS results from Fig. 1.

### Initial Stages of Oxidation by HOMB & The Protective Layer

**Figure 4:**
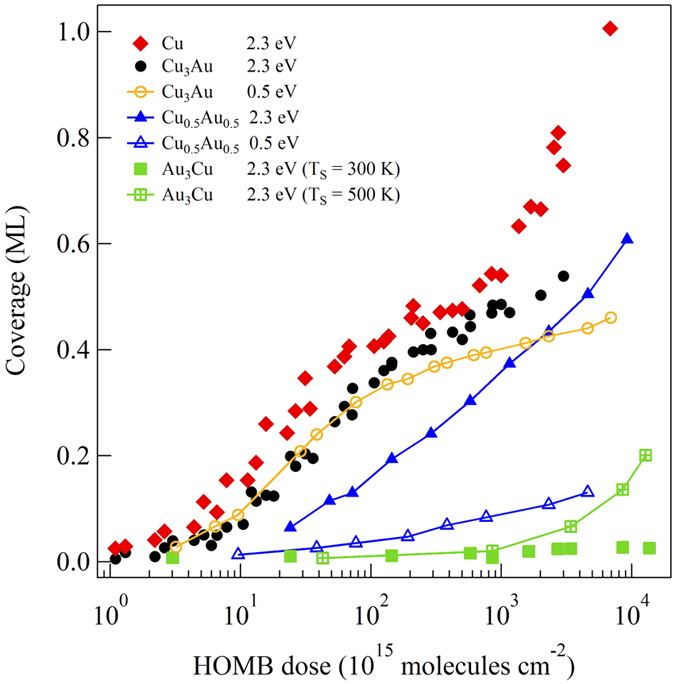
Oxygen (O) uptake curves for 2.3 eV hyperthermal O_2_ molecular beam (HOMB) incident at surface temperature *T*_S_ = 300 K on Cu(111) (red filled 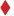), Cu_3_Au(111) (black filled 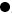), Cu_0.5_Au_0.5_ (111) (blue filled 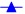), and Au_3_Cu(111) (green filled 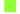); 0.5 eV HOMB incidence on Cu_3_Au(111) (orange 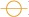) and Cu_0.5_Au_0.5_(111) (blue 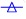). O uptake curves for 2.3 eV HOMB incident at surface temperature *T*_S_ = 500 K on Au_3_Cu(111) (green 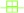), also shown. HOMB incident along the surface normal.

In Fig. 4 we show O-uptake curves obtained by integrating a series of O-1s spectra measurements taken after exposing Cu_3_Au(111), CuAu(111), and Au_3_Cu(111) to HOMB. The HOMB energies used, viz., *E*_HOMB_ = 2.3 eV and *E*_HOMB_ = 0.5 eV, correspond to 27000 K and 5900 K, respectively. On Cu_3_Au(111), the initial dissociative adsorption of O_2_ (O-coverage: ca. *Θ* ≤ 0.3 ML) does not depend on the incident energy. This indicates that an incident energy of 0.5 eV is enough to overcome the activation barrier for surface Cu-O formation. A previous study reported that a 0.6 eV HOMB would be more efficient to induce initial oxidation, as compared to a 2.3 eV HOMB, at the same nozzle temperature of ca. 1400 K. In the present study (0.5 eV HOMB), we used a nozzle temperature of ca. 300 K. Thus, the difference with previous results may be ascribed to vibrational excitations. At a nozzle temperature of 1400 K, O_2_ vibrational states *ν* = 1 and *ν* = 2 have populations of ca. 16% and 3%, respectively.

The difference in the O-uptake curves of 2.3 and 0.5 eV HOMB for ca. *Θ* ≥ 0.3 ML can be attributed to the repulsive interactions between pre-adsorbed O and the incoming O_2_, increasing the activation barrier to dissociative adsorption. Moreover, oxide formation on the Cu-rich sites via collision induced absorption (CIA) by energetic HOMB may also contribute to the difference. More importantly, oxidation proceeds accompanied by Cu segregation to the topmost layer. The oxidation induced by the 2.3 eV HOMB occurs less effectively on Cu_3_Au(111) than on Cu(111). This result suggests that Au atoms increase the activation barrier to dissociative adsorption.

As we would expect from the Au layer profile shown in Table 1, we find CuAu(111) and Au_3_Cu(111) less susceptible to oxidation as compared to Cu_3_Au(111) and Cu(111). On the Au_3_Cu(111), almost no oxidation occurs even for 2.3 eV HOMB. Thus, two (stable) layers of Au is enough to protect against oxidation. From the slopes, we estimate the initial O sticking probabilities to be as follows: *S*_0_ = 1.59 × 10^−2^ at *E*_HOMB_ = 2.3 eV on Cu(111); *S*_0_ = 6.35 × 10^−3^(6.20 × 10^−3^) at *E*_HOMB_ = 2.3 eV(0.5 eV) on Cu_3_Au(111); *S*_0_ = 1.87 × 10^−3^(3.82 × 10^−4^) at *E*_HOMB_ = 2.3 eV(0.5 eV) on CuAu(111); and *S*_0_ = 6.13 × 10^−5^ at *E*_HOMB_ = 2.3 eV on Au_3_Cu(111). At low *Θ*, we expect that the reaction rate would be determined by the atomic density of Cu (Au) on surface. Our experimental results (cf., Table 1) show 49%-Cu (51%-Au)^13^, 6%-Cu (94%-Au), and 0%-Cu (100%-Au) on clean Cu_3_Au(111), CuAu(111), and Au_3_Cu(111), respectively. Therefore, we estimate the Cu atomic density ratio of Cu_3_Au(111), CuAu(111), and Au_3_Cu(111) to Cu(111) to be 0.47, 0.05, 0, respectively. The sticking probability ratio of Cu_3_Au(111), CuAu(111), and Au_3_Cu(111) to Cu(111) are 4.0 × 10^−1^, 1.2 × 10^−1^, and 3.8 × 10^−3^, respectively, at 2.3 eV HOMB. The sticking probability ratio of CuAu(111) to Cu_3_Au(111) is 6.2 × 10^−2^, at 0.5 eV HOMB. These sticking probability ratios agree well with the surface Cu atomic density ratio, so that the initial stage of oxidation, i.e., O_2_ dissociative adsorption, depends on the top-layer Cu (Au) concentrations. We can also associate the difference in initial O sticking probability ratio to the location of the corresponding *d*-band center with respect to the Fermi level *E*_F_. The shallower the *d*-band center, the more accessible the electrons, and the stronger the binding with O. In the case of Cu-Au alloys, the *d*-band center of Cu_3_Au (111) is shallower than that of Au_3_Cu (111) and CuAu (111) (cf., Figs 2 and 3).

The CIA process is less effective in the oxidation of Cu_3_Au(111) than of Cu(111). The Au layer profile of Cu_3_Au(111) after the oxidation is estimated to be as follows: *x*_1_ = 0, *x*_2_ = 0.47, and *x*_3_ = 0.45^13^. The oxidation proceeds accompanied by Cu segregation to the topmost layer. The Au-rich second and third layers prevent the bulk from further oxidation. Similar analysis is performed for CuAu(111) (ca. *Θ* = 0.6 ML) oxidized by 2.3 eV HOMB (cf., Supplementary Information). The obtained Au layer profile is as follows: *x*_1_ = 0, *x*_2_ = 1.0, and *x*_3_ = 0.56. The Au-rich second and third layers work as a protective layer against bulk oxidation.

### Breaking the Protective Layer

**Figure 5:**
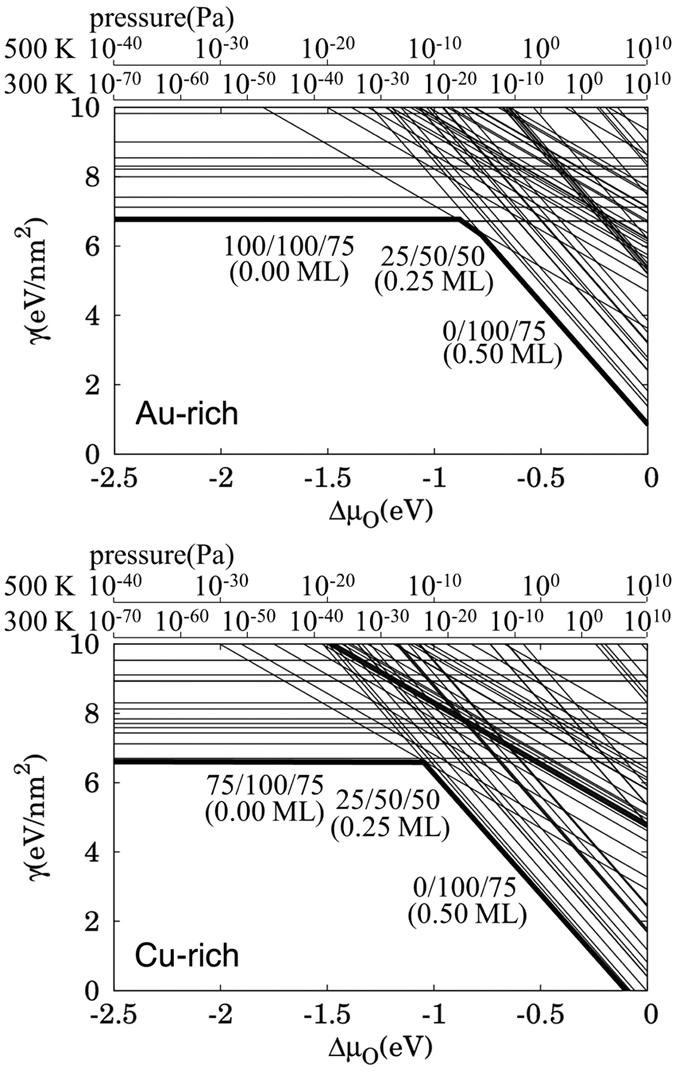
Surface free energy of Au_3_Cu(111) in equilibrium with Au-rich (upper panel) and Cu-rich (lower panel) Au_3_Cu bulk reservoir, as a function of the oxygen chemical potential Δ*μ*_o_ (which is also related to the oxygen partial pressures at 300 K and 500 K)^13^,^23^. Each line corresponds to one of the tested surface configurations. The lowest surface energy is the most stable and realized surface. The condition for perfect Au_3_Cu bulk is close to the Au-rich condition.

**Figure 6:**
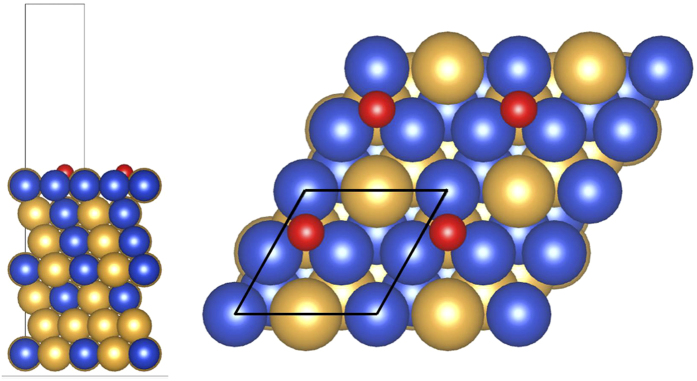
Optimized structure for O_0.25ML_/Au_3_Cu(111), with O adsorbed at the fcc-hollow site (cf., upper panel, Fig. 5, 500 K, 10^−8^ Pa). Au: yellow ball. Cu: blue ball. O: red ball. Structure drawn using the VESTA package^39^.

**Figure 7:**
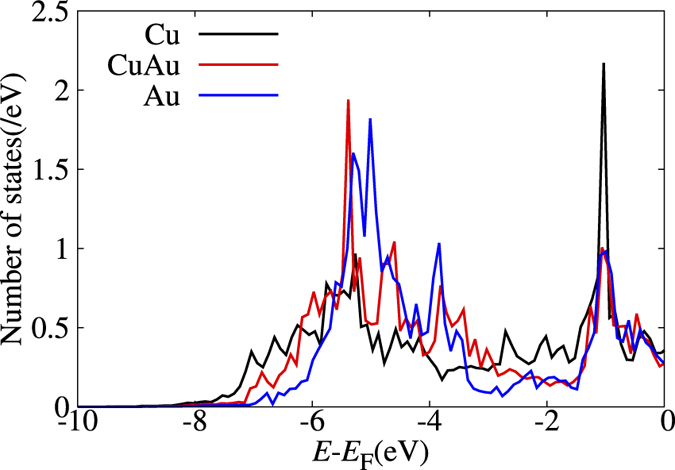
Projected density of states of adsorbed O on Cu (black line), CuAu(red line) and Au(blue line) at top layer. Other layers is the Au_3_Cu. Energies are given in [eV] with respect to the Fermi level (*E*_F_). (Calculated binding energy *E*_B_ = |*E − E*_F_|).

Here, we show how such a protective layer is broken. As mentioned above, Au_3_Cu(111), with a concentration profile of 100%-Au for the surface and subsurface layers, is impervious to oxidation by 2.3 eV HOMB. However, when we increase the surface temperature to 500 K, oxidation proceeds and we obtain an O coverage of *Θ* = 0.2 ML. Cu atoms segregate on the surface and the obtained Au layer profile is *x*_1_ = 0.31, *x*_2_ = 0.43, *x*_3_ = 0.51 (cf., Supplementary Information). In Fig. 5, we show the calculated surface energy of Au_3_Cu (111), under Au-rich and Cu-rich conditions, as a function of the oxygen chemical potential Δ*μ*_O_ (which is in turn related to the oxygen partial pressures at 300 K and 500 K). For the Au-rich case, the most stable clean surface has an Au layer profile of 100/100/75, i.e., Au on top and second layers, Au_3_Cu on third layer. For the Cu-rich case, the most stable clean surface has an Au layer profile of 75/100/75. The only difference with the Au-rich is the top layer, which is Au_3_Cu. The second and third layers are the same as that for the Au-rich case. With increasing chemical potential, both Au-rich and Cu-rich cases show the same tendency: O/Cu_3_Au on the top layer, CuAu in the second, and third layers; 2O/Cu on the top layer, with Au on the second and Au_3_Cu third layer. LEED shows a (2 × 2) pattern (cf., Supplementary Information), and we show the corresponding optimized surface structure in Fig. 6, with 0.25 ML of adsorbed O. The most stable O adsorption position is the fcc-hollow site, which is surrounded by Cu. The Cu-O distance is 0.188 nm. This distance does not depend on second and third layer configuration, and the value is close to Cu(111), CuAu(111) and Cu_3_Au(111). In Fig. 7, we show the corresponding projected density of states for the adsorbed O for three different Au surface (top layer) profiles, viz., 0, 50%, and 100% Au. As the surface becomes Au-rich, the bonding orbitals between Cu and O shift towards the Fermi level *E*_F_. This means that the richer the Au profile, the weaker the bonding between O and the surface. From above results, the *d*-band center, bonding orbitals, and the distance between Cu and O depend on configuration of top layer and does not depend on configuration of other layers and bulk component. From Fig. 6, at 500 K, we could expect small amounts of Cu atoms segregating to the topmost layer^2^ and enhancing the dissociative adsorption of O_2_.

## Conclusion

We determined, both experimentally (HOMB + SR-XPS) and theoretically (DFT-based calculations), the surface Au concentration profile of Cu-Au alloys (viz., Cu_3_Au, CuAu, and Au_3_Cu) in vacuum. We also studied the initial stages of oxidation of the corresponding surfaces. We observed Au segregation to the surface and subsurface of these Cu-Au alloys. The degree of segregation strongly depends on the bulk Au components. The richer the Au bulk components, the richer the Au surface segregation. The Au-rich layers form a protective layer against oxidation of the Cu-Au alloys. After exposing the corresponding surfaces to HOMB, we found that surfaces with higher concentrations of Au showed lower susceptibility to oxidation, as determined by the low O sticking probability. At 500 K, Cu segregates on the surface, breaking the protective layer, and oxidation proceeds on the surface, albeit rather slowly as there is still the subsurface. This gives further insight into how we can control the reactivity and robustness of a material, i.e., via the bulk component and the segregation profile.

## Additional Information

**How to cite this article**: Okada, M. *et al*. Experimental and Theoretical Studies on Oxidation of Cu-Au Alloy Surfaces: Effect of Bulk Au Concentration. *Sci. Rep.* **6**, 31101; doi: 10.1038/srep31101 (2016).

## Supplementary Material

Supplementary Information

## Figures and Tables

**Table 1:** The layer profile of Au atomic fraction (%) for Cu_3_Au, CuAu, and Au_3_Cu (111) surfaces.

(111) surface	%-Au@ 1^st^ layer	%-Au@ 2^nd^ layer	%-Au@ 3^rd^ layer	Ref.
Cu_3_Au	51	32	bulk (25)	13^a^
	50	25	25	13^b^
CuAu	94	72	bulk (50)	*^a^
	100	75	50	*^b^
Au_3_Cu	100	100	bulk(75)	*^a^
	100	100	75	*^b^

^a^Experimental mesurements.

^b^Theoretical calculations.

*Present work.
