# Supplementary material for: Experimental and Theoretical Studies on Oxidation of Cu-Au Alloy Surfaces: Effect of Bulk Au Concentration
Source: Sci Rep. 2016 Aug 12;6:31101. doi: 10.1038/srep31101 (PMC4981884; doi:10.1038/srep31101)
Supplement: Supplementary Information [file srep31101-s1.pdf]

Supplementary Information for

Experimental and Theoretical Studies on Oxidation of Cu-Au Alloy Surfaces —Effect of Bulk Au Concentration—

Michio Okada, Yasutaka Tsuda, Kohei Oka, Kazuki Kojima,
Wilson Agerico Diño, AkitakaYoshigoe, and Hideaki Kasai

Electron Inelastic Mean Free Path (IMFP)

We calculated the electron inelastic mean free path length (IMFP) using the method of Seah and Dench [S1]. Here, we show in detail the procedures for calculating the IMFP in Cu_3Au, CuAu, and Au_3Cu. For the materials considered in this study, we used the following expression for the IMFP, expressed in monolayer, i.e., λ_m

$$\lambda_m = \frac{538}{E^2} + 0.41(aE)^{1/2}. \tag{S1}$$

The kinetic energy of the electron E [eV] can be obtained from the Au-4f peak position. The monolayer thickness a [nm] can be obtained from the atomic or molecular weight A, the bulk density ρ, and the number of atoms in the molecules n, via the equation

$$a^3 = \frac{A}{\rho n N_A}, \tag{S2}$$

where N_A is Avogadro's number. Conversion of λ_m to the IMFP expressed in nm, i.e., λ_n, can be done via the equation

$$\lambda_n = a\lambda_m. \tag{S3}$$

For Cu_3Au, $A = 387.6$, $\rho = 12.3$ g $\cdot$ cm^{-3}, and $n = 4$.

From equations (S1) and (S3), we get $\lambda_m = 6.30$ and $\lambda_n = 1.48$ nm, respectively.

Similarly, for CuAu, with $A = 260.5$, $\rho = 14.9\ \mathrm{g \cdot cm^{-3}}$, and $n = 2$, we get $\lambda_\mathrm{n} = 1.56$ nm.

And again, for Au_3Cu, $A = 654.5$, $\rho = 17.2\ \mathrm{g \cdot cm^{-3}}$, and $n = 4$, we get $\lambda_\mathrm{n} = 1.63$ nm.

Au Layer Profile after Surface Oxidation

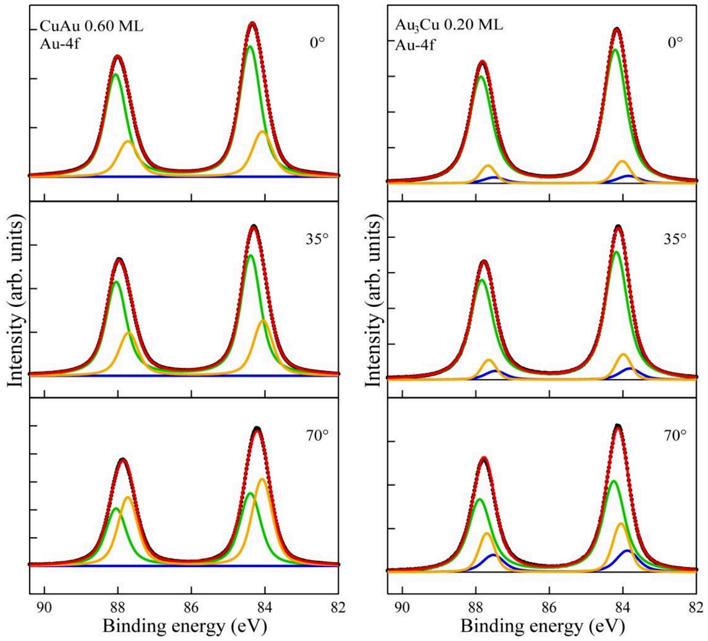

Figure S1: Detection angle dependence of Au-4f SR-XPS spectra on clean CuAu(111) at 0.60 ML (left panel) and $Au_3Cu(111)$ 0.20 ML (right panel). Surface normal detection: 0°. Oblique detection: 35° and 70°. The XPS spectra can be clearly separated into bulk (B), surface (S), and interface (I) components, corresponding to green, blue, and orange lines, respectively (see also text). The background was already subtracted by the Shirley method [S2]. Intensities given in arbitrary units and intensity scales differ between panels (i.e., differ between samples and detection angles).

Figure S1 shows the Au-4f SR-XPS spectra of CuAu(111) at $\Theta = 0.60$ ML and $Au_3Cu(111)$ at $\Theta = 0.20$ ML, measured at 0°, 35°, and 70° from the surface normal. We see that we can now separate both the Au-4f$_{7/2}$

and Au-4f$_{5/2}$ XPS peaks into three components, viz., the bulk (B) and surface (S) components, and an additional interface-layer (I) component. Here, we adopt the same peak shape and position for the S and B components for both the clean and oxidized surfaces. For CuAu(111) at Θ = 0.60 ML, the S component decreases in intensity with increasing Θ and disappears at Θ = 0.60 ML. At the same time, we observe a newly developed I component (CLS = -331 meV), which most probably comes from the Au atoms in the interface-layer, situated between the topmost oxidized Cu-O layer and the third (sub-surface) metallic bulk layer. Previous studies on $Cu_3Au(100)$ [S3, S4], $Cu_3Au(110)$ [S5, S6], and $Cu_3Au(111)$ [S7] also report observing similar I components. This suggests that, during HOMB irradiation, the Au-atoms in the top-layer become almost depleted due to the strong Cu segregation on the surface, as we have also observed on Cu_3Au.

We approximate the peak intensity ratio of I to B ($A_{\mathrm{I}}/A_{\mathrm{B}}$) using the following simple equation,

$$\begin{aligned}\frac{A_{\mathrm{I}}}{A_{\mathrm{B}}} &= \frac{x_2\ \mathrm{e}^{-(n-1)d/\lambda\cos\theta}}{\sum_{n=3}^{\infty} x_n\ \mathrm{e}^{-(n-1)d/\lambda\cos\theta}} \\ &= \frac{x_2}{x_3\ \mathrm{e}^{-d/\lambda\cos\theta} + \sum_{n=4}^{\infty} x_n\ \mathrm{e}^{-(n-2)d/\lambda\cos\theta}}. \end{aligned} \tag{S4}$$

x_n gives the Au fraction of the n-th layer from the surface. d gives the interlayer distance. The corresponding Au-4f photoelectron IMFP λ in each Cu-Au alloy can be obtained using the method discussed in Section above [S1]. θ is the photoelectron detection angle from the surface normal. From $A_{\mathrm{I}}/A_{\mathrm{B}}$ measured at $\theta = 0°$, $35°$, and $70°$, we obtained $x_1 = 0$, $x_2 = 1.0$, and $x_3 = 0.56$, assuming d to be the bulk interlayer distance, ignoring layer relaxation and taking $x_n \geq 4$ to be the bulk value.

For $Au_3Cu(111)$ at Θ = 0.20 ML, the surface was oxidized at a surface temperature setting of 500 K. Similar to CuAu(111), the B, S, and I components also appears on the Au-4f spectra for the oxidized $Au_3Cu(111)$. The CLS of I component is −193 meV. From $A_{\mathrm{I}}/A_{\mathrm{B}}$ measured at $\theta = 0°$, $35°$, and $70°$, we obtained $x_2 = 0.43$ and $x_3 = 0.51$. Also, we obtained $x_3 = 0.31$ from the peak intensity ratio of S to that of the clean surface.

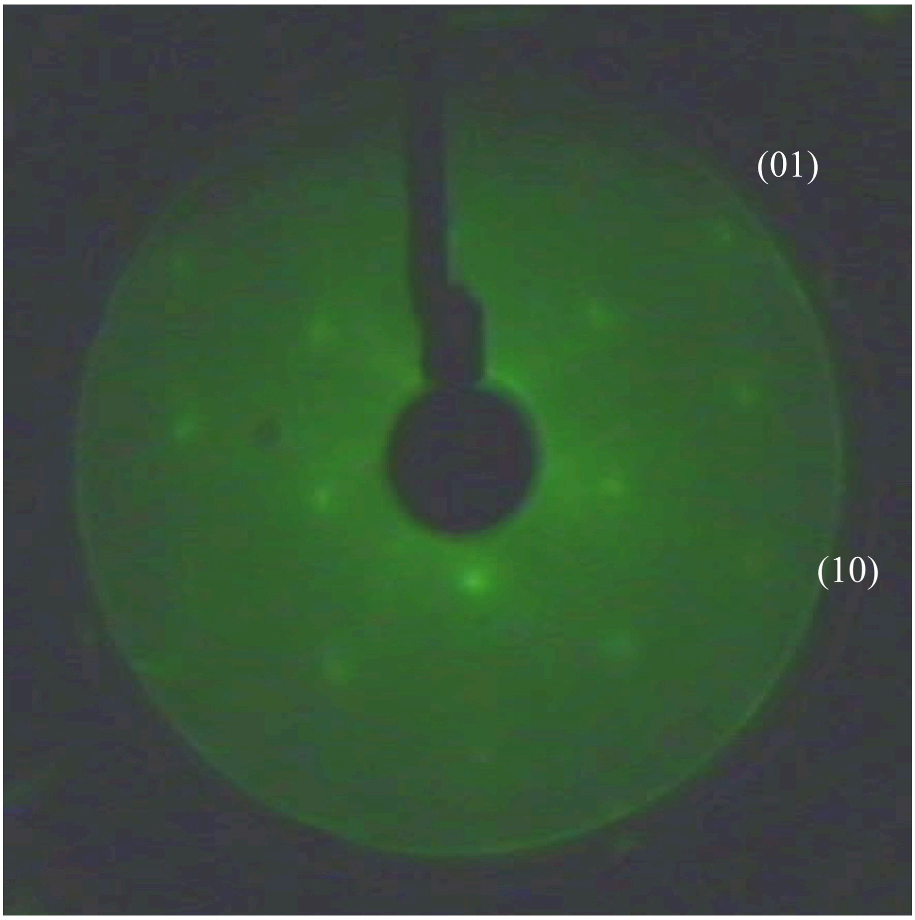

Figure S2: (2×2) LEED pattern of $O_{0.20\ \mathrm{ML}}/Au_3Cu(111)$ at 500 K. Electron beam energy $E_\mathrm{p} = 83.5$ eV.

LEED Pattern of $O_{0.20\ \mathrm{ML}}/Au_3Cu(111)$ prepared at 500 K

Figure S2 shows a (2×2) LEED pattern for $O_{0.20\ \mathrm{ML}}/Au_3Cu(111)$ at 500 K.

Surface Energy

Following some detailed thermodynamic derivations found in the literature (cf., e.g., [S8, S9, S7, S10, S11]), we address the surface segregation of clean and oxidized Cu-Au alloys, viz., CuAu(111) and $Au_3Cu(111)$, by calculating the corresponding surface free energy γ (using the slab model) given by

$$\gamma = \frac{1}{S^{\mathrm{slab}}}[G^{\mathrm{slab}} - \sum_i \mu_i N_i]. \tag{S5}$$

S^{slab} is the surface area of the slab. G^{slab} is the Gibbs free energy of the slab. μ_i is the chemical potentials of each atomic species i ($i = \mathrm{Cu, Au, O}$). N_i are the number of each atomic species i in the slab. Assuming that the alloy surface is in equilibrium with the underlying bulk reservoir, we have

$$\mu^{\mathrm{bulk}} = x_{\mathrm{Au}}^{\mathrm{bulk}} \Delta\mu_{\mathrm{Au-Cu}} + \mu_{\mathrm{Cu}}, \tag{S6}$$

where

$$\Delta\mu_{\mathrm{Au-Cu}} = \mu_{\mathrm{Au}} - \mu_{\mathrm{Cu}}. \tag{S7}$$

x_i^{bulk} is the mole fraction of the atomic species i ($i = \mathrm{Cu, Au}$) in the Cu-Au alloys. For example, for Au_3Cu, $x_{\mathrm{Cu}}^{\mathrm{bulk}} = 0.25$ and $x_{\mathrm{Au}}^{\mathrm{bulk}} = 0.75$. As an estimate, the range of $\Delta\mu_{\mathrm{Au-Cu}}$ spans the two limits corresponding to the phase separation of Cu (Cu-rich limit, when the bulk reservoir is rich in Cu) and the phase separation of Au (Au-rich limit, when the bulk reservoir is rich in Au). From equation (S7)

$$\Delta\mu_{\mathrm{Au-Cu}}^{\mathrm{Cu-rich}} \leq \Delta\mu_{\mathrm{Au-Cu}} \leq \Delta\mu_{\mathrm{Au-Cu}}^{\mathrm{Au-rich}}, \tag{S8}$$

where

$$\Delta\mu_{\mathrm{Au-Cu}}^{\mathrm{Cu-rich}} = \frac{\mu^{\mathrm{bulk}} - \mu_{\mathrm{Cu}}^{\mathrm{fcc}}}{1 - x_{\mathrm{Cu}}^{\mathrm{bulk}}} \tag{S9}$$

and

$$\Delta\mu_{\mathrm{Au-Cu}}^{\mathrm{Au-rich}} = \frac{\mu_{\mathrm{Au}}^{\mathrm{fcc}} - \mu^{\mathrm{bulk}}}{1 - x_{\mathrm{Au}}^{\mathrm{bulk}}}. \tag{S10}$$

$\mu_{\mathrm{Cu}}^{\mathrm{fcc}}$ and $\mu_{\mathrm{Au}}^{\mathrm{fcc}}$ are the chemical potentials of Cu and Au in the fcc bulk, respectively.

Similarly, assuming that the alloy surface is in equilibrium with the surrounding gas phase (O_2 at temperature T and partial pressure p_{O_2}), we have

$$\mu_{\mathrm{O}} = \frac{1}{2}\mu_{\mathrm{O}_2}^{\mathrm{gas}}(T, p_{\mathrm{O}_2}) \tag{S11}$$

and

$$\mu_{\mathrm{O}_2}^{\mathrm{gas}}(T, p_{\mathrm{O}_2}) = E_{\mathrm{O}_2}^{\mathrm{gas}} + 2\Delta\mu_{\mathrm{O}}(T, p_{\mathrm{O}_2}), \tag{S12}$$

$E_{\mathrm{O}_2}^{\mathrm{gas}}$ is the total energy of an isolated O_2 at $T = 0$ K, which gives the upper limit of $\mu_{\mathrm{O}_2}^{\mathrm{gas}}$. Assuming an ideal gas, the second term on the right hand side of equation (S12) depends on the difference in chemical potential of O_2 at $T = 0$ K and the temperature of interest T, at the reference pressure p°, i.e.,

$$\Delta\mu_{\mathrm{O}}(T, p_{\mathrm{O}_2}) = \Delta\mu_{\mathrm{O}}(T, p^{\circ}) + \frac{1}{2}k_{\mathrm{B}}T\ln\left(\frac{p_{\mathrm{O}_2}}{p^{\circ}}\right), \tag{S13}$$

where

$$\begin{aligned}\Delta\mu_{\mathrm{O}}(T, p^{\circ}) = {} & \frac{1}{2}[H_{\mathrm{O}_2}(T, p^{\circ}) - H_{\mathrm{O}_2}(0\ \mathrm{K}, p^{\circ})] \\ & -\frac{1}{2}T[S_{\mathrm{O}_2}(T, p^{\circ}) - S_{\mathrm{O}_2}(0\ \mathrm{K}, p^{\circ})].\end{aligned} \tag{S14}$$

k_{B} is the Boltzmann constant. $H_{\mathrm{O}_2}(T, p^{\circ})$ and $S_{\mathrm{O}_2}(T, p^{\circ})$ are the enthalpy and entropy of O_2 at the temperature T and reference pressure p°, respectively [S12]. Finally, we can recast equation (S5) into the following form

$$\begin{aligned}\gamma = \frac{1}{S^{\mathrm{slab}}}[& G^{\mathrm{slab}} - N^{\mathrm{slab}}\mu^{\mathrm{bulk}} - N^{\mathrm{slab}}\Delta\mu_{\mathrm{Au-Cu}}(x_{\mathrm{Au}}^{\mathrm{slab}} - x_{\mathrm{Au}}^{\mathrm{bulk}}) \\ & -\frac{N_{\mathrm{O}}}{2}E_{\mathrm{O}_2}^{\mathrm{gas}} - N_{\mathrm{O}}\Delta\mu_{\mathrm{O}}(T, p_{\mathrm{O}_2})].\end{aligned} \tag{S15}$$

N^{slab} and $x_{\mathrm{Au}}^{\mathrm{slab}}$ are the total number of metal atoms and the mole fraction of Au in the slab of the Cu-Au alloys, respectively. N_{O} is the number of O atoms in the slab.

References

[S1] Seah, M. P. & Dench, W. A. Quantitative electron spectroscopy of surfaces: a standard data base for electron inelastic mean free paths in solids. *Surf. Interface Anal.* **1**, 2-11 (1979).

[S2] Shirley, D. A. High-resolution X-ray photoemission spectrum of the valence bands of gold. *Phys. Rev.* B **5**, 4709-4714 (1972).

[S3] Okada, M. et al. Protective layer formation during oxidation of $Cu_3Au(100)$ using hyperthermal O_2 molecular beam. *Appl. Phys. Lett.* **89**, 201912-1-3 (2006).

[S4] Okada, M. et al. Comparative study of oxidation on Cu and Cu_3Au surfaces with a hyperthermal O_2 molecular beam. *Surf. Sci.* **600**, 4228-4232 (2006).

[S5] Okada, M. & Teraoka, Y. Active oxidation of $Cu_3Au(110)$ using hyperthermal O_2 molecular beam. *Appl. Surf. Sci.* **256**, 5676-5680 (2010).

[S6] Hashinokuchi, M. Yoshigoe, A. Teraoka, Y. & Okada, M. Temperature dependence of Cu_2O formation on $Cu_3Au(110)$ surface with energetic O_2 molecular beams. *Appl. Surf. Sci.* **287**, 282-286 (2013).

[S7] Tsuda, Y. et al. Initial stages of $Cu_3Au(111)$ oxidation: oxygen induced Cu segregation and the protective Au layer profile. *Phys. Chem. Chem. Phys.* **16**, 3815-3822 (2014).

[S8] Zangwill, A. *Physics at Surfaces* (Cambridge University Press, Cambridge, 1988).

[S9] Desjonquères, M. -C. Spanjaard, D. *Concepts in Surface Physics 2nd edn* (Springer-Verlag, Berlin, 1996).

[S10] Kitchin, J. R. Reuter K. & Sheffler, M. Alloy surface segregation in reactive environments: first-principles atomistic thermodynamics study of $Ag_3Pd(111)$ in oxygen atmospheres. *Phys. Rev.* B **77**, 075437-1-12 (2008).

[S11] Reuter, K. & Sheffler, M. Composition, structure, and stability of $RuO_2(110)$ as a function of oxygen pressure. *Phys. Rev.* B **65**, 035406-1-11 (2001).

[S12] *JANAF Tables.* Available at: http://kinetics.nist.gov/janaf/ (Last accessed: December 2015).
